# Measurement and Analysis of Root Anchorage Effect on Stalk Forces in Lodged Corn Harvesting

**DOI:** 10.3389/fpls.2022.852375

**Published:** 2022-04-12

**Authors:** Qiankun Fu, Jun Fu, Zhi Chen, Chao Chen, Jialiang Zhang, Luquan Ren

**Affiliations:** ^1^College of Biological and Agricultural Engineering, Jilin University, Changchun, China; ^2^Key Laboratory of Bionics Engineering, Ministry of Education, Jilin University, Changchun, China; ^3^Chinese Academy of Agricultural Mechanization Sciences, Beijing, China

**Keywords:** root anchorage, lodged corn, corn harvest, harvester header, bending moment, static torque sensor

## Abstract

The effect of root anchorage on corn stalk is the main cause of difficulties in stalk lifting and ear picking of lodged corn. To quantify the forces on the stalks caused by root anchorage in corn harvesting, a root force measurement system was designed and applied in this study. The bending moment and torsional moment on the upright and lodged corn stalks were measured in corn harvesting with the designed system and the results were compared with the manually measured failure boundaries. The manually measured results showed bending moments to push down the upright stalks, to lift the lodged corn stalks, and to slip the lodged corn stalks were 35.12, 23.33, and 40.36 Nm, respectively, whereas the torsional moments needed to twist off the upright and lodged corn stalks were 4.02 and 3.33 Nm, respectively. The bending moments that the corn header applied to the upright, forward lodged, reverse lodged, and lateral lodged corn stalks were 10.68, 22.24, 16.56, and 20.42 Nm, respectively, whereas the torsional moments on them were 1.32, 1.59, 1.55, and 1.77 Nm, respectively. The bending force was the main factor that broke the root anchorage and influenced the stalk movement of lodged corn in harvesting. By analyzing the bending moment curves on the lodged corn stalks, it was proposed that for the harvesting of corn lodged in the forward, reverse, and lateral direction, the corresponding harvester header improvement suggestions are enlarging the size of pins on the gathering chains, reducing the speed of gathering chains, and lengthening the snouts with a sleeker surface, respectively. This study provides base data for the root anchorage effect on lodged corn and provides references for the improved design of the corn harvester header.

## Introduction

Corn lodging is usually caused by excessive planting density, improper use of fertilizer, unreasonable irrigation or diseases, and pests during the growth period (Echezona, 2007; Shelby et al., 2018; Liu et al., 2021). The bending strength and rind puncture resistance of stalks are usually taken as the indices for lodging resistance in variety breeding (Albrecht et al., 1986; Robertson et al., 2016; Seegmiller et al., 2020). The main effect of lodging is to reduce crop yield (Ma et al., 2014). The lodging area statistics and yield reduction prediction are usually carried out with satellite remote sensing and UAV images (Han et al., 2018; Chauhana et al., 2019; Wilke et al., 2019). Lodging in the corn mature period such as the late milky ripeness stage and the wax ripeness stage is usually caused by extreme weather such as heavy rains and rainstorms. This kind of lodging is characterized as whole plant inclination because of the loosening of root-soil (Martinez-Vazquez, 2016). Lodging in the corn maturation period causes severe ear loss in harvesting (Wang et al., 2021) because the harvester would not be able to pick corn ears lower than the working height of the corn header (Paulsen et al., 2014; Xue et al., 2020a). Sugarcane has the same lodging morphology as corn stalk. The lodged sugarcane is lifted with the spiral dividers in the harvest (Bai et al., 2021). Short-stem crops such as wheat and rice are harvested with lowered headers to cut off the crop and feed the whole plant to the threshing part (Paulsen et al., 2014; Phetmanyseng et al., 2019).

**FIGURE 1:**
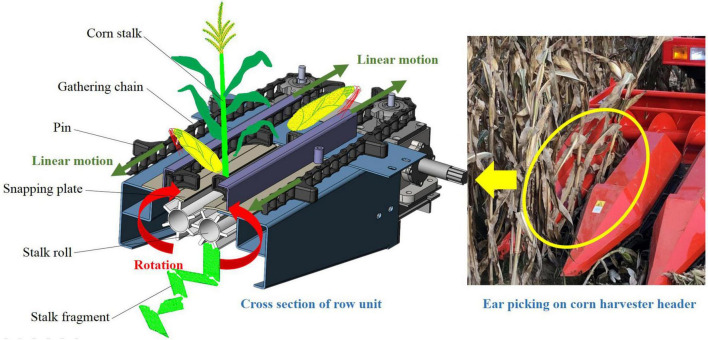
Process of ear picking on the corn header.

Different from the harvest of sugarcane and the short-stem crops, the stalk is not cut in corn harvesting. Lowering header height and applying headers with narrow row spacing units are the main compromise means in the lodged corn harvesting (Yang et al., 2016; Wang et al., 2021). In the harvesting, the snouts of the header are extended into the bottom of the stalks to lift them with the travel of the harvester. The stalks are fed into the gap between the snapping plates and pulled down by the stalk rolls under the snapping plates. Then the corn ears are picked by the blocking effect of the snapping plates, as shown in Figure 1. In this process, the corn stalk is not only subjected to forces by the stalk rolls and the pins on the gathering chains but also influenced by the root anchorage (Fu et al., 2019). In the previous studies, Donovan et al. (1982) measured the pulling force of the corn root with a sensor connected to the three-point linkage of the tractor. Reneau et al. (2020) tested the contribution of brace roots on anchorage by measuring the deflection forces after removing them. However, there is still a lack of reliable data about the root anchorage effect on the lodged corn stalks in corn harvesting. The technical difficulties such as stalk lifting, stalk feeding, and header blockage could not be solved without reliable analysis. It is also impossible to make a reasonably improved design on corn harvester header in dealing with lodged corn.

Bending failure and torsional failure are the main forms of stalk failure in lodged corn harvesting. The bending moment and torsional moment on the stalks could be taken as the indices for the root anchorage in corn harvesting (Francisco et al., 2018; Stubbs et al., 2019). This study focused on the forces acting on upright and lodged corn stalks affected by the root anchorage in the lodged corn harvesting. It starts with the measurement of the bending moment and torsional moment to cause stalk failure on the upright and lodged corn stalks. Then the bending moment and torsional moment on the upright and lodged corn stalks exerted by the corn header were measured in the field test with a designed measurement system. The force analysis was made on the stalks by comparing the test results with the failure boundaries. The action laws of corn header on the upright stalks, forward lodged corn stalks, reverse lodged corn stalks, and lateral lodged corn stalks were obtained with the analysis of bending moment curves on corn stalks. The causes of ear miss picking and header blockage were discussed, and suggestions for corn header improved design in lodged corn harvest were propounded. The results of this study will give base data for the root anchorage effect on lodged corn and provide references for the improved design of the corn harvester header.

## Materials and Methods

### Mechanical Properties of Corn Stalks

The mechanical properties of corn stalk are the physical basis for the stress analysis of stalk in corn harvesting. The parameters measured in this test were the force required to push down the upright stalks, the lifting force of the lodged corn stalks, the slipping force of the lodged corn stalks, and the torsional moments to twist off of upright and lodged corn stalks. The tested variety in this study was Xianyu 335, which was widely planted in Northeast China. The test was made in Changpaozi Village, Yitong Manchu Autonomous County, Siping City, Jilin Province. The corn lodged in 3 typhoons between Aug 27 and Sep 8, 2020, about 30–40 days before the test.

#### Pushing Down Force of the Upright Corn

**FIGURE 2:**
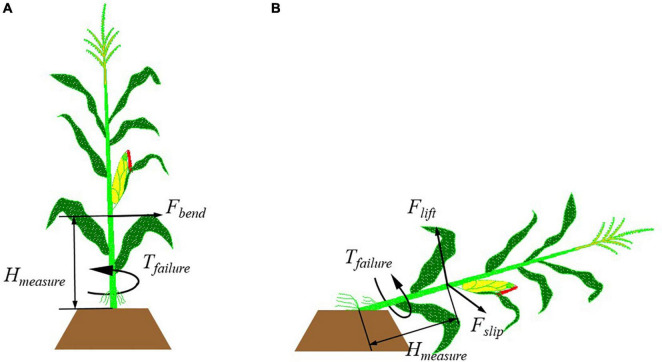
Measurement method of the mechanical properties of upright and lodged corn stalk. **(A)** *F*_*bend*_ and *T*_*failure*_ on the upright stalks; **(B)** *F*_*lift*_, *F*_*slip*_, and *T*_*failure*_ on the lodged corn stalks.

A hand-held dynamometer was used to measure the forces. The dynamometer was an HP-300 model with a relative error of 0.5%, which was manufactured by Yueqing Handpi Instruments Co., Ltd. A horizontal force was applied to the upright corn stalks with the dynamometer until the corn was pushed down to the ground. This maximum force value in pushing down the upright corn stalk was recorded. The stalk failure might occur at the root of corn when the soil loosened or at any position below the force application position when the stalk broke. The measurement method is shown in Figure 2A. To compare the measured force with the forces in corn harvesting, the force *F*_*bend*_ was applied at the height of 400 mm above the ground, which was approximately equal to the minimum working height of the corn header according to the harvester operating instructions. The height was approximate to the force application height of the corn header to corn stalks. So were the heights *H*_*measure*_ in the measurement of *F*_*lift*_ and *F*_*slip*_ in the following tests. The bending moments on the stalks were obtained with the product of the force and the height.

#### Lifting Force of Lodged Stalk

In the harvest of lodged corn, the snout tips of the corn header extend into the stalks’ bottom, the pins on the gathering chains lift the stalks with corn ear to the corn header’s working height. Therefore, the stalk lifting force of lodged corn stalks was measured. The measurement method is shown as *F*_*lift*_ in Figure 2B. The maximum force was recorded until the ear was lifted to the height of 500 mm. This height was determined according to the working height of the applied corn harvester header, which was 400 mm. The bending moments were obtained with the product of *F*_*lift*_ and *H*_*measure*_.

#### The Slipping Force of Lodged Corn Stalks

When the lodged corn stalk is subjected to a lateral force, it may slide on the ground. Since the corn root is fixed by the soil, and the top of the corn stalk can be regarded as a free end, when the lodged corn stalk is subjected to a horizontal force, the corn stalk would slide around the root on the ground. This sliding will cause the stacking of stalks in front of the corn header and cause corn header blockage eventually. It should be avoided in corn harvesting. Therefore, the critical condition for the occurrence of stalk slipping was measured. The measurement method of this force is shown as *F*_*slip*_ in Figure 2B.

#### Torsional Moment Boundaries of Corn Stalk Failure

Torsion is also considered an important reason for stalk failure (Faisal et al., 2017). In this test, a torsional force was applied to the upright and lodged corn stalks to get the failure boundary of the stalks under the torsional moment. The torsional moment was recorded as *T*_*measure*_ when the stalk was twisted off or the root is ripped out from the soil. The measurement method on the upright and lodged corn stalks is shown in Figures 2A,B. The torsional moment was applied and measured with a torque wrench (PLARZ-30 Nm by Suzhou Duotong Hardware Electrical Co., Ltd.). The stalks were clamped with a fixture on the top of the torque wrench. The abrasive cloth was wrapped around the corn stalk to increase friction force because the friction between the stalk and the steel clamping fixture was too small to prevent relative sliding. When the stalks got lodged, the torsional forces could be applied to any position of the stalks by the harvester header snouts. To reduce the interference of stalk elastic deformation on the measurement results, and avoid the influence of brace roots on stalk clamping, the measurement was made at the height of 150 mm above the ground.

### Forces on the Stalks in Corn Harvesting

**FIGURE 3:**
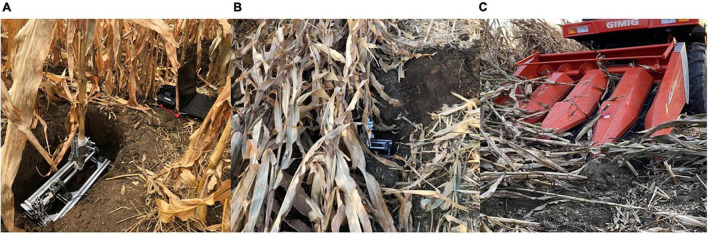
An experiment of forces on stalks in corn harvesting. **(A)** Experiment on the upright corn; **(B)** experiment on the lodged corn; **(C)** the experiment scene of corn harvest.

In this section, the torsional moment and bending moment on the upright and lodged corn stalks in harvesting were measured. According to the relationship between corn lodging direction and harvester travel direction, the lodged corn stalks were classified into forward, reverse, and lateral, corresponding to the harvester traveling in the same, opposite, and vertical direction with stalk lodging. The harvester applied in the experiment was the 4YZP-4Y corn harvester manufactured by Juming Company in Shandong, China. The speed of the harvester was reduced to 0.5 m/s to meet the operation requirements of lodged corn, as its standard working speed was 0.55–1.1 m/s according to the operating instructions. In the measurement, the measured corn plant was fixed on the mounting frame. The mounting frame was placed in an installation pit. It ensured that the measured corn plant was at the same height with natural growth and avoided the collision between the harvester and the mounting frame in harvesting. Figure 3 shows the scene of the field experiment.

#### Measurement System

**FIGURE 4:**
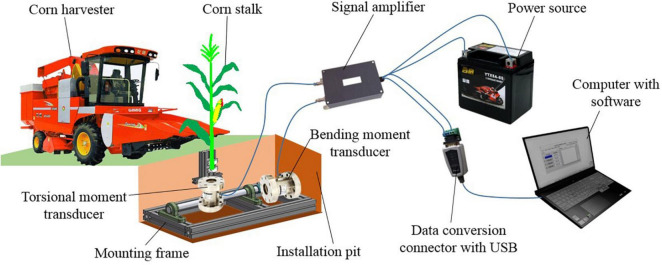
Measurement of forces on corn stalks in harvesting.

Figure 4 shows the measurement system for the forces on the stalk in corn harvesting. The system consisted of a corn harvester, an installation pit to place the mounting frame, a mounting frame with a torsional moment transducer and bending moment transducer, a whole corn plant fixed on the mounting frame, a signal amplifier, a data conversion connector with USB, a computer with acquisition software, and a 12 V power source. The power of the sensors and the signal amplifier were supplied by a 12 V power source. The data were displayed on the acquisition software of the computer after format transformation.

**FIGURE 5:**
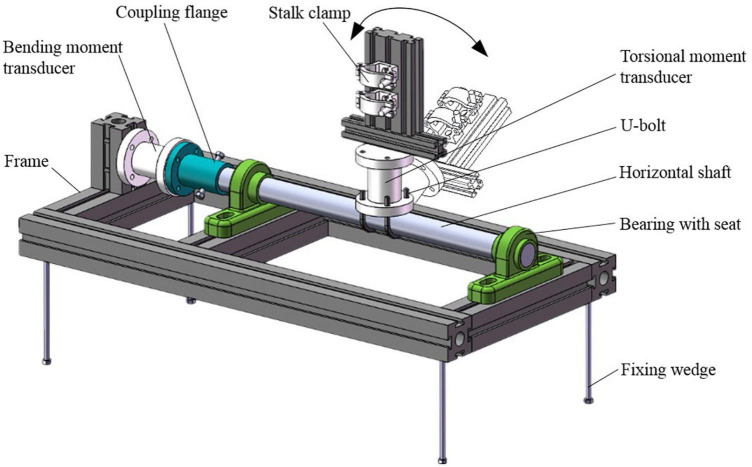
Structural diagram of the mounting frame.

The structure of the mounting frame is shown in Figure 5. It was assembled from profiles. The upper part of the mounting frame was a stalk clamp to fix the stalk. At the bottom of the stalk clamp was the torsional moment transducer (ZNNF-5 Nm static torque sensor by Bengbu Zhongnuo Sensor Co., Ltd.). When the stalk was twisted, the torque signal would be collected by the torsional moment transducer. The torsional moment transducer was fixed on the horizontal shaft with U-bolts. It could rotate around the horizontal shaft to simulate the morphology of the upright and lodged corn stalks at different angles. The horizontal shaft was installed on the frame through bearings with the seat. The bending moment transducer (ZNNF-100 Nm static torque sensor by Bengbu Zhongnuo Sensor Co., Ltd.) was connected with the horizontal shaft at one end and fixed with the frame at the other end. When the stalk was subjected to forces by the harvester, the bending moments would be collected in the form of static torque. There were four fixing wedges at the bottom of the frame. They could be stuck into the soil to prevent frame movement.

#### Measurement Method

**FIGURE 6:**
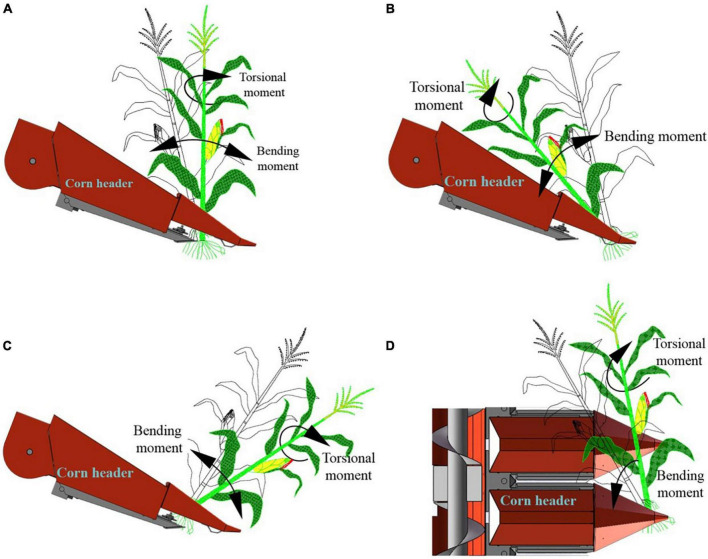
Measurement methods of forces on upright and lodged corn stalks. **(A)** Upright stalks; **(B)** forward lodged stalks; **(C)** reverse lodged stalks; **(D)** lateral lodged stalks.

The tests were conducted on upright and lodged corn. The bending moments in the harvester traveling direction and the torsional moments were measured on the upright corn, as shown in Figure 6A. In the harvest of forward and reverse lodged corn, the bending moments in the harvester traveling direction and the torsional moments on the stalks were collected, as shown in Figures 6B,C. When the harvesting was lateral to the corn lodging direction as Figure 6D, the bending moments in the stalk lifting direction and the torsional moments on the stalks were measured. Each measurement was repeated 60 times.

## Results and Discussion

### Properties of the Upright and Lodged Corn Stalks

#### Force Bearing Capacities

**FIGURE 7:**
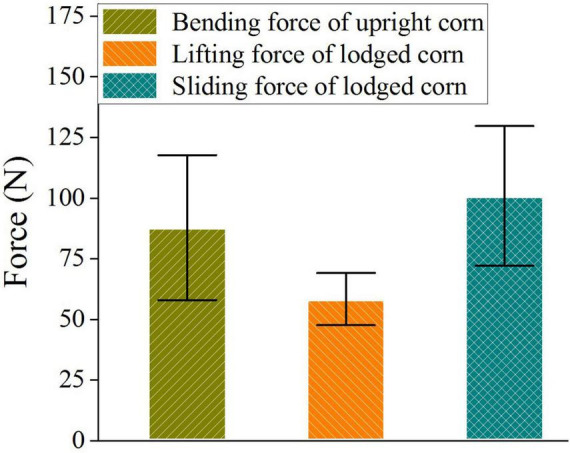
Force-bearing capacities of upright and lodged corn stalks. Error bars indicate standard deviations.

Figure 7 shows the maximum forces that the upright and lodged corn stalks could bear under different force conditions. The average force to push down the upright corn stalks was 87.80 ± 29.89 N, the average lifting force of the lodged corn stalks was 58.33 ± 10.76 N, and the average slipping force of lodged corn stalks was 100.91 ± 28.79 N. The bending moments on the stalks under the three situations were 35.12, 23.33, and 40.36 Nm, respectively, supposing the effects of the adjacent stalks not considered. The failure bending moment of the upright stalks was larger than Sekhon et al. (2020) measured with the equipment of DARLING on corn 40 days after anthesis, but it was close to Xue et al. (2020b)’s result measured at the ear position after physical maturity. The difference might result from the varieties, growth stages, and the stalk conditions caused by planting patterns. The upright stalks usually broke at the bottom internodes. It showed that the anchoring effect of the root was strong, just as Donovan et al. (1982) verified in the test. It was measured that the pulling force of the root varied between 836 and 1767 N. However, it was much easier to lift the lodged corn stalks than push down the upright stalks. Because the soil structure was destroyed in lodging and the fixation of soil on the root decreased greatly in the lodging direction. The force to slip the lodged corn stalks was much larger. It could be ascribed to the anchorage of root in the slipping direction, even though it was weakened in the lifting direction. This phenomenon was proved in the anchorage of brace roots by Reneau et al. (2020). The force to slip lodged stalk was a little larger than that to push down the upright stalk because the stacking of lodged stalks restricts the slipping.

#### Failure Torsional Moment of Corn Stalks

**FIGURE 8:**
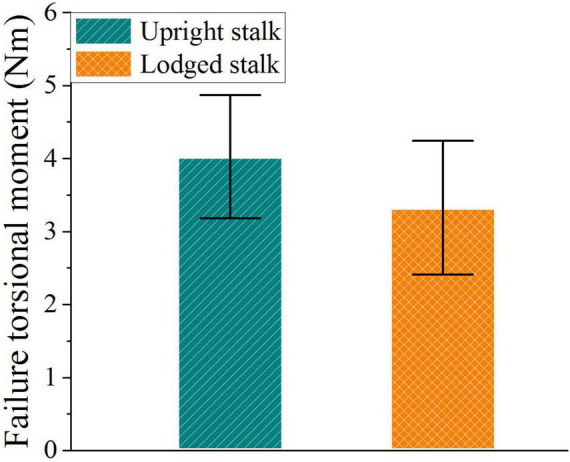
Failure torsional moment of upright and lodged corn stalks. Error bars indicate standard deviations.

The torsional moment required to twist the upright and lodged corn stalks is shown in Figure 8. The average failure torsional moment of the upright stalks was 4.02 ± 0.84 Nm, and that of lodged corn stalks was 3.33 ± 0.92 Nm. The weaker torsional bearing capacity of lodged corn stalks might be attributed to the decreasing of moisture content because of the insufficient supply of water after lodging, just like the declining of stalk strength after corn maturity (Xue et al., 2020b). Additionally, the destruction of soil also made the root easier to rotate under torsional force. Windsor-Collins et al. (2007) and Faisal et al. (2017) had measured the torsional force of palm petals and plant petioles similarly in the previous studies. But a few data were recorded on the torsional moment of corn stalks, which may be because the clamping devices could not apply sufficient friction on the sleek surface of the corn stalk. The problem was solved with the wound abrasive cloth between the clamping device and the corn stalks in this experiment.

### Effect of Root Anchorage on the Stalk in Corn Harvesting

#### Bending Moment and Torsional Moment on Corn Stalk

**FIGURE 9:**
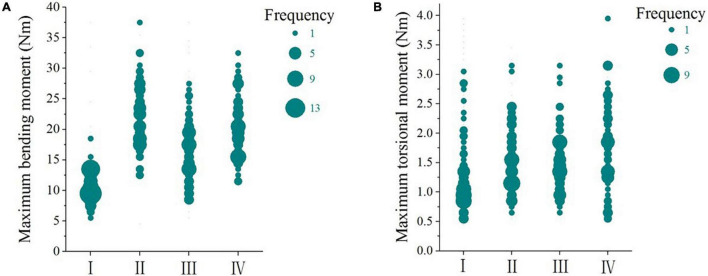
Frequency distribution of bending moments and torsional moments on stalks in harvesting. **(A)** Bending moments; **(B)**, torsional moments; I upright stalks; II forward lodged stalks; III, reverse lodged stalks; IV, lateral lodged stalks.

The maximum and minimum values of bending moments and torsional moments on the stalks in corn harvesting are shown in Table 1, and the distributions of maximums are plotted in Figure 9. For the bending moment on the upright stalks and the torsional moments on all stalks, the direction in which the maximum bending moments appeared was specified as positive. But for the lodged corn stalks, the stalk lifting direction was specified as positive.

**TABLE 1:** Bending moments and torsional moments on stalks in corn harvesting (Nm).

	Maximum bending moment	Minimum bending moment	Maximum torsional moment	Minimum torsional moment
Upright stalks	10.68 ± 2.35	−3.01 ± 2.87	1.32 ± 0.61	−0.26 ± 0.15
Forward lodged stalks	22.24 ± 5.27	−6.25 ± 4.16	1.59 ± 0.56	−0.37 ± 0.20
Reverse lodged stalks	16.56 ± 4.75	−4.82 ± 4.30	1.55 ± 0.55	−0.32 ± 0.25
Lateral lodged stalks	20.42 ± 4.96	−4.40 ± 3.65	1.77 ± 0.74	−0.50 ± 0.39

The bending moments on the lodged corn stalks were greater than that on the upright stalks, which indicated larger forces on the lodged corn. If the bending force was in the same direction with corn lodging, it would push the stalks to the ground. If it was opposite to the lodging direction, it would lift the stalks.

The maximum bending moments and torsional moments on the upright stalks were approximately 30% to the stalks’ failure boundaries. Figure 9A shows that the bending moments of the upright stalks varied in a small range. The speed calculations in corn header design ensured that the upright stalks would not be pushed down by the harvester header in harvesting (Wang et al., 2016).

When the harvester traveled in the same direction with corn lodging, the stalks were forced backward by the pins on the gathering chains of the corn header. Table 1 shows that the average bending moment on the stalks was 22.24 Nm, which was near to the bending moment calculated with the lifting force of the lodged corn stalks. When the harvester traveled opposite to the corn lodging direction, the average bending moment on the corn stalks was 16.56 Nm, which was at approximately two-thirds to the lifting moment of the lodged corn stalks. Figure 9A indicates many of the tested stalks were not lifted. When the harvester traveled lateral to the corn lodging direction, the average bending moment on the stalks was 20.42 Nm. It can be inferred that most of the stalks were lifted when the header snouts extended into the stalk bottom.

It can be seen from Figure 9B that the bending moments on lodged corn stalks did not show a definitive increase compared to the upright stalks. These bending moments were around half of the stalk failure torsional moment and could not fracture the stalks in corn harvesting. But the stalks were more susceptible to failure when the bending load was combined with torsional moment (Stubbs et al., 2019).

#### Bending Moment Curves Analysis

**FIGURE 10:**
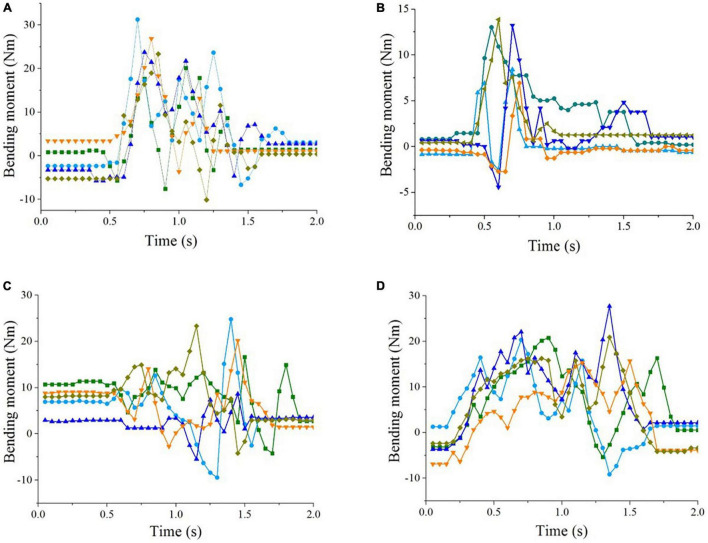
Typical stalk bending moment curves under tested harvesting conditions. **(A)** Upright stalks; **(B)** forward lodged stalks; **(C)** reverse lodged stalks; **(D)** lateral lodged stalks.

The bending was the main cause of stalk failure in corn harvesting. The typical bending moment curves on the stalks under the tested working conditions are shown in Figure 10.

In the harvest of upright corn (Figure 10A), the ear picking was completed in a short time. The bending moments reached the maximum shortly after the harvest began. For the forward lodged corn stalks (Figure 10B), the bending moment peak values were larger than that of the upright corn. The bending moments reached the maximum in a short time and declined with fluctuations with the ear picking. Contrarily, in the harvesting of reverse lodged corn stalks, the bending moments were small at the beginning and got larger gradually with fluctuations (Figure 10C). Figure 10D shows the bending moments on the lateral lodged corn stalks in harvesting. The stalks were lifted by the inclined surface of snouts with harvester traveling. The bending moments increased gradually at the beginning and fluctuated later.

The bending moment curves on the harvested corn stalks showed that the corn header has different operation characteristics for corn in upright and different lodging states. The operation characteristics depended on the structural and kinematic parameters of the corn header. The harvesting capacity of the corn header on lodged corn can be improved by optimizing the parameters of the harvester header.

#### Inspirations From the Bending Moment Curves

**FIGURE 11:**
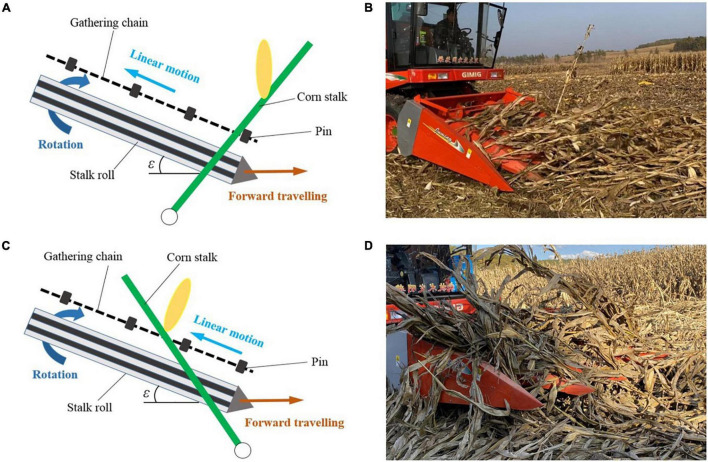
Corn harvested in the forward and reverse directions of lodging. **(A)** Motion relationship between corn header and forward lodged corn stalks; **(B)** corn harvested smoothly in the forward direction; **(C)** motion relationship between corn header and reverse lodged corn stalks; **(D)** header blockage in the harvesting of reverse lodged corn.

The contrast between the bending moment curves on the forward and reverse lodged corn stalks reflected the differences in force applied by the corn header. For the forward lodged corn, the forces were first applied to the lower part of a stalk, then the whole stalk was lifted by the pins on the gathering chain, as Figure 11A. The stalk was pulled upright in a short time. The stalk pulling and ear picking could be completed smoothly (Figure 11B). To improve the efficiency of stalk lifting and avoid ear loss caused by miss-lifting, the size of pins on the gathering chains should be enlarged. And the pins on both sides had better be arranged symmetrically rather than misplaced.

Forces on the reverse lodged corn were applied from the top of the stalks. Due to the stalk deformation and the anchorage of the root, the stalks could not be lifted and fed into the feeding space of the corn header rapidly as shown in Figure 11C. The ear was prone to be miss picked, and the header was prone to be blocked, as shown in Figure 11D. This result differed from the conclusions of Xue et al. (2020a) that the lowest loss occurred when the corn was harvested in the reverse direction of lodging. The above difference may be caused by the simultaneous presence of stalk breaking and root lodging in their test, which disturbed stalk feeding in the forward and lateral directions caused severe header blockage.

In fact, in the feeding of upright corn, the velocity of the stalks could be regarded as equal to the absolute horizontal velocity of pins on the gathering chains. To avoid the stalks being pushed forward by the corn header, the absolute velocity of the pins on the gathering chains should be in the opposite direction with harvester traveling, as shown in the following formula:


(1) V=pinahVharvester-Vpincosε<0

Where *V*_*pinah*_ was the absolute horizontal velocity of the pin, *V*_*harvester*_ was the travel velocity of the harvester, *v*_*pin*_ was the linear motion velocity of pins driven by the gathering chains, and ε was the inclination angle of corn header.

But in the ear picking of reverse lodged corn, it needs to push the stalks forward to lift them. The forces on the stalks should be applied in the forward direction of the harvester. The absolute velocities of the pins on the gathering chains should be in the same direction with the harvester traveling:


(2) $$V_{pinah}=V_{harvester}-V_{pin}\cos\varepsilon >0$$

The formulas (1) and (2) revealed why the corn header blockage and severe losses occurred when the harvester worked in the opposite direction with stalk lodging. In the harvest of sugarcane, the highest loss rate occurred when it was harvested opposite to the lodging direction (Tamaki et al., 2009). Because corn is harvested for the ears rather than the stalks, the corn stalks need to be pulled down by the stalk rolls after feeding. Therefore, the forces applied to the stalks on the corn header have a greater impact on corn harvesting (Zhang et al., 2021). Theoretically, to improve the corn header’s ear picking adaptability for the reverse lodged corn, the velocity of pins on the gathering chains should be largely reduced to help with the lifting and feeding of stalks. The speed-adjustable gathering chains on the corn header would be a feasible method for reverse lodged corn harvest.

**FIGURE 12:**
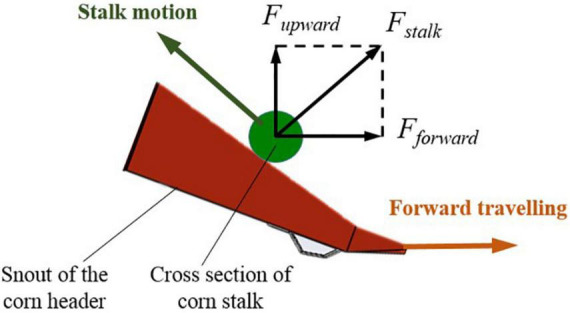
Forces on the lateral lodged corn stalks in the lifting.

In the harvesting of the lateral lodged corn stalks, a forward component force was applied to the stalk to slip it horizontally. Stalk slipping in front of the corn header may cause stalk stacking and header blockage. The component forces on a stalk are shown in Figure 12. The forces satisfied the following relationship:


(3) $$F_{forward}=F_{upward}\tan\theta$$

Where *F*_*forward*_ was the component force in the harvester forward direction, *F*_*upward*_ was the component force to lift the stalk, θ was the inclination angle of the snout surface, which was smaller than 30° (Chinese Academy of Agricultural Mechanization Sciences, 2007). It could be calculated that the *F*_*forward*_ was smaller than half of the *F*_*upward*_, and smaller than the stalk’s slipping force. The stalk would not slide on the ground as long as it is not stuck on the snout surface.

Therefore, to reduce header blockage caused by stalk stacking in lateral lodged corn harvesting, the following requirements should be satisfied in the header improved design. The inclination angle of the snouts should be less than the friction angle between snouts and the stalks; the tips of the snouts should be tapering and long enough to help it enter the stalk’s bottom; irregular shapes should be removed from the snout surface to avoid stalk stacking.

## Conclusion

In corn harvesting, the torsional moments applied to the upright and lodged corn stalks in the forward, reverse, and lateral directions by the corn harvester header were 1.32, 1.59, 1.55, and 1.77 Nm, respectively, much smaller than the torsional failure boundaries 4.02 and 3.33 Nm of the upright stalks and lodged corn stalks, respectively. The bending moments applied to the upright stalks and lodged corn stalks in the forward, reverse, and lateral directions were 10.68, 22.24, 16.56, and 20.42 Nm, respectively, while the moments to push down the upright stalks, to lift the lodged stalks, and to slip the lodged stalks were 35.12, 23.33, and 40.36 Nm, respectively. The bending force was the main factor that broke the root anchorage on the corn stalks and influenced stalk lifting.

Enough bending moment applied to the stalk was an important prerequisite for lodged corn stalks lifting and corn ear picking. To improve the ability of the corn header in lifting the lodged corn stalks by applying bending moments, suggestions for improvement were made corresponding to the stalks lodged in the forward, reverse, and lateral directions. In the harvesting of forward lodged corn, the size of pins on the gathering chains needed to be enlarged to improve the corn header feeding efficiency. For the reverse lodged corn, to avoid gathering chains applying forces opposite to the stalk lifting direction, it was necessary to slow down the speed of the gathering chains to accelerate stalk lifting. In the harvesting of lateral lodged corn, lengthening and tapering the snouts and sleeking the snouts surface could help them enter the stalk bottom smoothly and avoid header blockage.

## Data Availability Statement

The original contributions presented in the study are included in the article/supplementary material, further inquiries can be directed to the corresponding author.

## Author Contributions

QF: conceptualization, methodology, data curation, and writing—original draft preparation. JF: conceptualization, resources, writing—review and editing, funding acquisition, and formal analysis. ZC: resources, methodology, software, and project administration. CC and JZ: validation, data curation, writing—review and editing, and project administration. LR: formal analysis and proofreading. All authors have read and agreed to the published version of the manuscript.

## Conflict of Interest

The authors declare that the research was conducted in the absence of any commercial or financial relationships that could be construed as a potential conflict of interest.

## Publisher’s Note

All claims expressed in this article are solely those of the authors and do not necessarily represent those of their affiliated organizations, or those of the publisher, the editors and the reviewers. Any product that may be evaluated in this article, or claim that may be made by its manufacturer, is not guaranteed or endorsed by the publisher.
